# Parsing human and biophysical drivers of coral reef regimes

**DOI:** 10.1098/rspb.2018.2544

**Published:** 2019-02-13

**Authors:** Jean-Baptiste Jouffray, Lisa M. Wedding, Albert V. Norström, Mary K. Donovan, Gareth J. Williams, Larry B. Crowder, Ashley L. Erickson, Alan M. Friedlander, Nicholas A. J. Graham, Jamison M. Gove, Carrie V. Kappel, John N. Kittinger, Joey Lecky, Kirsten L. L. Oleson, Kimberly A. Selkoe, Crow White, Ivor D. Williams, Magnus Nyström

**Affiliations:** 1 Stockholm Resilience Centre, Stockholm University, Stockholm, Sweden; 2 Global Economic Dynamics and the Biosphere Academy Programme, Royal Swedish Academy of Sciences, Stockholm, Sweden; 3 Stanford Center for Ocean Solutions, Stanford University, Stanford, CA 94305, USA; 4 Hawai‘i Institute of Marine Biology, University of Hawai‘i at Mānoa, Kaneohe, HI 96744, USA; 5 School of Ocean Sciences, Bangor University, Anglesey LL59 5AB, UK; 6 Hopkins Marine Station, Stanford University, Pacific Grove, CA 9395, USA; 7 Pristine Seas, National Geographic Society, Washington, DC 20036, USA; 8 Lancaster Environment Centre, Lancaster University, Lancaster LA1 4YQ, UK; 9 Ecosystem Science Division, Pacific Islands Fisheries Science Center, National Oceanic Atmospheric Administration, Honolulu, HI, 96818, USA; 10 National Center for Ecological Analysis and Synthesis, University of California Santa Barbara, Santa Barbara, CA 93101, USA; 11 Center for Oceans, Conservation International, Honolulu, HI 96825, USA; 12 Julie Ann Wrigley Global Institute of Sustainability, Arizona State University, Tempe, AZ 85281, USA; 13 Department of Natural Resources and Environmental Management, University of Hawai‘i at Mānoa, Honolulu, HI 96822, USA; 14 Department of Biological Sciences, California Polytechnic State University, San Luis Obispo, CA 93407, USA

**Keywords:** boosted regression trees, ecology, Hawai‘i, interactions, management, regime shift

## Abstract

Coral reefs worldwide face unprecedented cumulative anthropogenic effects of interacting local human pressures, global climate change and distal social processes. Reefs are also bound by the natural biophysical environment within which they exist. In this context, a key challenge for effective management is understanding how anthropogenic and biophysical conditions interact to drive distinct coral reef configurations. Here, we use machine learning to conduct explanatory predictions on reef ecosystems defined by both fish and benthic communities. Drawing on the most spatially extensive dataset available across the Hawaiian archipelago—20 anthropogenic and biophysical predictors over 620 survey sites—we model the occurrence of four distinct reef regimes and provide a novel approach to quantify the relative influence of human and environmental variables in shaping reef ecosystems. Our findings highlight the nuances of what underpins different coral reef regimes, the overwhelming importance of biophysical predictors and how a reef's natural setting may either expand or narrow the opportunity space for management interventions. The methods developed through this study can help inform reef practitioners and hold promises for replication across a broad range of ecosystems.

## 1. Introduction

Coral reef ecosystems worldwide are shifting to alternative regimes, driven by a combination of human impacts, biotic processes and abiotic conditions [1,2]. Beyond abrupt changes in ecosystem structure and function [2], long-lasting regime shifts may bear heavy costs to society through the loss of ecosystem services associated with a particular regime [3]. They also pose serious challenges for coral reef managers [4], since reversing undesirable regimes can be difficult and costly owing to strong reinforcing feedback mechanisms [5,6].

To date, descriptions of alternative reef regimes have predominantly addressed benthic community structure, with an emphasis on shifts from coral to algal dominance [7–9]. Changes in fish assemblages have also been highlighted, either as a driver of benthic regime shifts [10] or as their direct consequence [11]. Given the strong interdependence between benthic and fish communities on coral reefs however, disentangling ‘what drives what’ becomes problematic. Recent work by Donovan *et al*. [12] addresses this issue by proposing a broader approach that combines both fish and benthic functional groups as the defining elements of reef regimes. Such an integrated description of the reef community provides a more nuanced view of reef regimes which better captures the complexity of coral reef dynamics. Yet, what drives the occurrence of these integrated regimes and how to subsequently prioritize management actions remain unknown.

In the face of escalating human impacts, such as overfishing, reduced water quality and effects from climate change, there is growing awareness surrounding the multi-causality of reef regimes [8] and potential effects of interacting stressors [13,14]. Effectively managing coral reefs therefore requires an accurate, and often context-specific, understanding of how multiple drivers combine to support or undermine different regimes. In particular, discerning the relative influence of anthropogenic versus biophysical drivers is critical to appreciate how environmental conditions might limit or favour different management options. Although humans can become the dominant force determining coral reef ecosystem state [15], variations in biophysical drivers, such as waves and primary productivity, set natural bounds on ecosystem condition even in the absence of local human influence [16,17].

The main Hawaiian Islands—the populated portion of the Hawaiian archipelago, hereafter referred to as the Hawaiian Islands for brevity—span gradients in both environmental conditions [18] and human pressures [19], allowing for an exploration of their relative importance in determining the spatial distribution of reef regimes. The Hawaiian Islands are also the focus of one of the most extensive spatial databases of biophysical and anthropogenic predictors available for a coastal ecosystem [20]. Here, we use this database to predict the occurrence of multiple reef regimes defined by both fish and benthic communities. We apply boosted regression trees to quantify the relative influence of each biophysical and anthropogenic predictor, identify relationships between predictors and regimes, and characterize interaction patterns. Identifying what predicts different reef ecosystem regimes and how the natural environment can influence management opportunities is essential to help practitioners effectively anticipate, avoid and respond to coral reef change.

## 2. Methods

### (a) Study area and reef regimes

**Figure 1.**
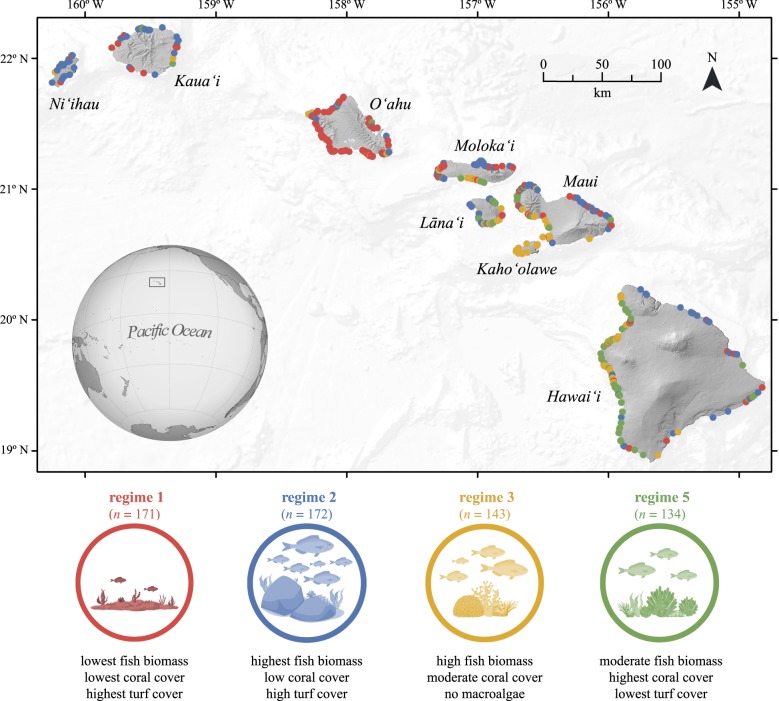
Map of the study area showing the location of 620 sites across the main Hawaiian Islands (Hawai‘i, USA), categorized into four distinct reef regimes. Key characteristics of each regime are provided below the respective icons. Explore an interactive version of the map at https://stanford.maps.arcgis.com/apps/StoryMapBasic/index.html?appid=b50b97f3cadb4c919a85bb6e4dd654cd.

Situated in the middle of the Pacific Ocean, the Hawaiian Islands consist of eight high volcanic islands with varying human population density and exposure to natural forces [19]. The study builds on data from more than 1000 forereef habitat sampling locations (i.e. reef slope habitat exposed to the open ocean) across the region that were recently classified into five reef regimes using model-based clustering of 10 fish and benthic functional groups (electronic supplementary material, table S1). Each cluster is a mixture of multivariate distributions composed of the densities of each component (i.e. fish and benthic functional groups), and each observation is assigned to a cluster based on the probability of membership given the observation [12]. Out of the five regimes, however, Donovan *et al*. [12] identified one as a highly variable and transitional state (i.e. regime 4). Given the ambition to accurately associate predictors to the spatial occurrence of distinct regimes, we removed the sites classified into regime 4 to reduce noise in the data and optimize predictive performance. We also excluded the 25% most uncertain classifications (i.e. sites with the lowest probability of being classified again into the same regime), thereby retaining 620 sites most representative of four distinct reef regimes (figure 1; electronic supplementary material, table S1), and hereafter referred to as regime 1, 2, 3 and 5 for consistency with Donovan *et al*. [12].

Sites classified into regime 1 show the characteristics of a degraded reef, i.e. low fish biomass, low hard coral cover and high algae cover (electronic supplementary material, table S1). Regime 2 is characterized by rugose habitat with high fish biomass (e.g. browsers such as *Kyphosus hawaiiensis* and *Naso unicornis*), high turf and macroalgae cover, and low hard coral cover. Regime 3 exhibits high fish biomass and turf algae cover, no macroalgae and moderate hard coral cover. Regime 5 displays moderate fish biomass, less turf algae and higher hard coral cover, commonly comprised of the coral *Porites compressa*. For detailed methodology and description of the regimes, see Donovan *et al*. [12].

### (b) Anthropogenic and biophysical predictors

We reviewed all continuous spatial layers of anthropogenic and environmental drivers compiled by Wedding *et al*. [20] for coastal waters of the Hawaiian Islands and retained a set of 20 predictors (table 1; electronic supplementary material, table S2) based on ecological relevance (electronic supplementary material, table S3) and collinearity analysis (electronic supplementary material, figure S1 and table S4). We used pairwise relationship correlation coefficients (no coefficient greater than |0.6|) and variance inflation factor estimates (scores lower than 3.5) to assess collinearity among predictors.

**Table 1.** Predictor variables used to explain the occurrence of multiple reef regimes. (See the electronic supplementary material, table S2 for extended descriptions. Raster data can be visualized in an online map viewer at http://www.pacioos.hawaii.edu/projects/oceantippingpoints/#data.)

	predictor	description	temporal range	spatial resolution (m)
anthropogenic	effluent	nutrient run off (gallon/day/7 km^2^) from onsite waste disposal systems (cesspools and septic tanks)	2009–2014	500
	sedimentation	estimate of annual average amount of sediment (tons yr^−1^) delivered offshore	2005	100
	new development	relative level (0 to 1) of new development along the coastline	2005–2011	100
	habitat modification	presence-absence of any alteration or removal of geomorphic structure as a result of human use	2001–2013	500
	invasive algae	observed presence of any invasive algae	2000–2013	500
	commercial fishing	annual average commercial reef fisheries catch (kg ha^−1^)	2003–2013	100
	non-commercial boat fishing	annual average non-commercial boat-based reef fisheries catch (kg ha^−1^) from all gear types	2004–2013	100
	non-commercial shore fishing_line	annual average non-commercial shore-based reef fisheries catch (kg ha^−1^) by line	2004–2013	100
	non-commercial shore fishing_net	annual average non-commercial shore-based reef fisheries catch (kg ha^−1^) by net	2004–2013	100
	non-commercial shore fishing_spear	annual average non-commercial shore-based reef fisheries catch (kg ha^−1^) by spear	2004–2013	100
biophysical	SST _max	maximum monthly climatological mean of sea surface temperature (°C)	1985–2013	5000
	SST_STD	standard deviation of the long-term mean of weekly sea surface temperature (°C)	2000–2013	5000
	chlorophyll_max	maximum monthly climatological mean of chlorophyll-*a* (mg m^−3^)	2002–2013	4000
	chlorophyll_anomaly	annual average of the total number of anomalous events for chlorophyll-*a*	2002–2013	4000
	irradiance_max	maximum monthly climatological mean of photosynthetically available radiation (Einstein m^−2^ d^−1^)	2002–2013	4000
	irradiance_STD	standard deviation of the long-term mean of 8 days irradiance composites (Einstein m^−2^ d^−1^)	2002–2013	4000
	wave_max	maximum monthly climatological mean of wave power (kW m^−1^)	1979–2013	500–1000
	wave_anomaly	annual average of the total number of anomalous events for wave power	2000–2013	500–1000
	complexity	topographical complexity of the seafloor measured as slope of slope (i.e. the maximum rate of change in seafloor slope)	1999–2000	5
	depth	depth of the seafloor in metres	1999–2000	5

The selection of anthropogenic predictors expanded on a human dimensions framework that identified the primary human impacts mediating coral reef condition [21]. It includes catch from commercial and non-commercial fisheries, land-based stressors (effluent, sedimentation, new development), habitat modification and invasive species [20]. Non-commercial fisheries were further characterized by platform (boat- versus shore-based) and gear types (line, net, spear). Gear types were combined for non-commercial boat-based fisheries to account for collinearity.

Biophysical predictors were derived from time series of variables known to be major drivers of coral reef ecosystems: sea surface temperature, chlorophyll-*a* (as a proxy for phytoplankton biomass and thus primary production), irradiance and wave power. Five climatological metrics were available for each predictor: long-term mean, standard deviation of the long-term mean, maximum monthly climatological mean, maximum anomaly and frequency of anomalies [18,20]. Choices were made to eliminate highly correlated metrics (electronic supplementary material, figure S2), while ensuring for each predictor that both the actual forcing and its variability were represented. We used the maximum monthly climatological mean (i.e. the largest value of the 12 monthly climatological values averaged over more than 10 years) to represent the actual forcing since spatial variations in ecological communities are largely defined by their climatological envelope as communities tend to adapt to the extremes in the seasonal cycle [18]. Depending on collinearity (electronic supplementary material, figure S2), either the standard deviation of the long-term mean or the frequency of anomalous events (i.e. the percentage of time above the maximum monthly climatological mean) was used to capture environmental stability, or lack thereof. The depth and topographical complexity of the seafloor, derived from high-resolution bathymetry of the region, were also included owing to their well-known importance in structuring reef communities (electronic supplementary material, table S3).

For a majority of the datasets, the temporal range represented approximately a 10 year average, which matched the temporal spread of the biological surveys used to identify regimes [12] and provided an estimate of long-term trends in spatial gradients rather than a single snapshot in time. For detailed methodology on each anthropogenic and biophysical predictor raster, see Wedding *et al*. [20].

### (c) Data analyses

All statistical analyses were conducted using R v. 3.3.2 [22]. Statistical scripts and custom R package *ggBRT* are available on GitHub (https://github.com/JBjouffray). We used boosted regression trees (BRTs) [23] to examine the occurrence of each regime in relation to anthropogenic and biophysical predictors. BRTs represent an advanced regression technique that combines large numbers of relatively simple trees by sequentially fitting each new tree to the residuals from the previous ones. It improves predictive performance over more traditional tree fitting techniques with the ability to fit non-linear relationships and account for complex interactions among predictors [23].

The classification of sites into different regimes was converted to presence-absence of each regime [8] and modelled using a Bernoulli distribution following the *gbm.step* routine [23] in the *dismo* package v. 1.1-4 [24]. Trees were built with default parameters to make model outputs comparable among regimes: a tree complexity of 5, a learning rate of 0.001 and a bag fraction of 0.75. Tree complexity controls how many levels of interactions are fitted, learning rate determines the contribution of each new tree to the model and bag fraction specifies the proportion of data to be randomly selected while fitting each single decision tree [23,25]. Variation of these parameters by running all possible combinations of tree complexity (1–5), learning rate (0.01, 0.005, 0.001, 0.0001) and bag fraction (0.5, 0.7, 0.9) provided negligible improvements in predictive performance.

Model performance was evaluated by 10-fold cross-validation that allows to test the model against withheld portions of the data which are not used in model fitting [23]. We looked at the cross-validated per cent deviance explained, calculated as (1 – (cross-validated deviance/mean total deviance)) and cross-validated area under the receiver operating characteristics curve (AUC) as measures of model performance. An AUC value of 0.5 corresponds to a predictive ability similar to what would be expected by chance alone. Values are considered ‘acceptable’ between 0.7–0.8, ‘excellent’ between 0.8–0.9 and ‘outstanding’ above 0.9 [26]. Spatial autocorrelation was assessed by estimating Moran's *I* coefficients from the model residuals [27].

We calculated the relative importance of each predictor based on the number of times a variable was selected for splitting, weighted by the squared improvement to the model as a result of each split and averaged over all trees [23,28]. To assess the relative contribution of anthropogenic versus biophysical predictors for each regime, we considered only the variables with a relative influence above that expected by chance (100/number of variables, i.e. 5%) [29] and rescaled their influence to 100%.

Partial dependency plots with 95% confidence intervals obtained from 1000 bootstrap replicates [25] were used to visualize the relationships between the most influential predictor variables and the response (regime), while keeping all other predictors at their mean. We quantified relative interaction strength between predictors by measuring residual variation between pairwise model predictions with and without interactions [30]. We used 100 bootstrap resampling to test the significance of the strongest interactions. For each bootstrap, we randomly resampled the occurrence of the regime before re-fitting the BRT model and then recorded the size of the interactions to generate a distribution under the null hypothesis of no interaction among predictors [30].

Input data for the predictor variables had different native spatial resolutions (table 1). For instance, while many of the anthropogenic predictor rasters were available at a fine spatial grain (less than 500 m), most of the biophysical ones were generated at a coarser grain size (e.g. 4000 m). To control for the influence of different grain sizes on the outcome of the model, we extracted all predictor raster datasets at multiple standardized grain sizes (500, 1000, 1500, 2500 and 4000 m), before re-running the BRTs on regimes aggregated following a two-thirds majority within each cell resolution (electronic supplementary material, figure S3).

## 3. Results

### (a) Relative influence of human and biophysical predictors

**Figure 2.**
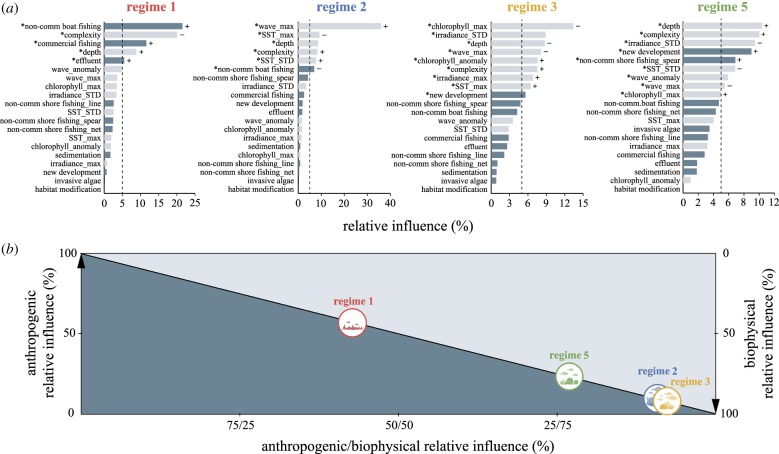
(*a*) Relative influence of anthropogenic (dark grey) and biophysical (light grey) predictor variables used to model the occurrence of each reef regime. The ‘asterisks’ mark variables with an influence above what could be expected by chance (greater than 5%, indicated by the dotted line). The signs + and − display the general direction of the relationship, when discernible. (*b*) Distribution of the four regimes along a continuum of anthropogenic versus biophysical relative contribution, calculated by considering only the variables with a relative influence greater than 5%. SST, sea surface temperature; max, maximum monthly climatological mean; STD, standard deviation of the long-term mean; anomaly, frequency of anomalies. (Online version in colour.)

BRT models performed well for all four regimes (electronic supplementary material, table S5), with deviance explained from 37% to 41%, high predictive performance (AUC scores between 0.88–0.91) and minimal spatial autocorrelation (Moran's *I* between 0.02 and 0.04). The pattern of predictors' contributions differed among regimes, with regimes 1 and 2 displaying a few strongly influential predictors, while regimes 3 and 5 were best explained by a broader, but less influential, set of variables (figure 2*a*). This was also reflected by the number of predictors having a relative influence above what could be expected by chance: five for regime 1, six for regime 2 and nine for regimes 3 and 5 (figure 2*a*). The regimes distributed along a continuum of biophysical and anthropogenic influence (figure 2*b*), with an overwhelming contribution of biophysical variables in predicting the occurrence of regimes 3, 2 and 5 (92%, 91% and 77% biophysical relative influence, respectively). Regime 1, on the other hand, was most effectively predicted by anthropogenic variables (57%).

### (b) Predicting the occurrence of reef regimes

**Figure 3.**
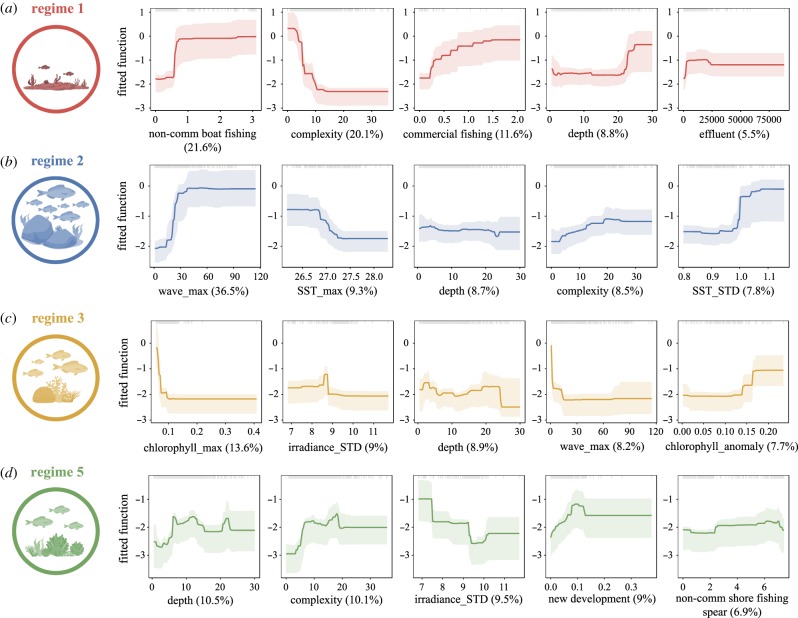
Partial dependency plots with 95% confidence intervals for the five most influential variables predicting the occurrence of four distinct reef regimes (*a*–*d*). The graphs show the effect of a given predictor on the probability of occurrence of the regime while keeping all other variables at their mean. Relative influence of each predictor is reported between parentheses. Grey tick marks across the top of each plot indicate observed data points. SST, sea surface temperature; max, maximum monthly climatological mean; STD, standard deviation of the long-term mean; anomaly, frequency of anomalies. (Online version in colour.)

For each regime, the relationships of the five most influential predictors (figure 3) and two strongest pairwise interactions (table 2; electronic supplementary material, figure S4) are described below. The probability of occurrence of regime 1 was higher as both non-commercial boat fishing catch (21.5% relative influence) and commercial fishing catch (11.6%) increased (figure 3*a*). Topographical complexity of the seafloor was the second strongest predictor (20.1%), suggesting regime 1 is more likely to occur in areas with low structural complexity. Depth (8.8%) and effluent (5.5%) both displayed positive relationships. Interaction patterns reflected the influence of the most important predictors with the probability of regime 1 occurring being greatest when fishing catch was high and structural complexity was low (table 2; electronic supplementary material, figure S4a). The model explained 41% of the deviance and had an AUC score of 0.90.

**Table 2.** Pairwise interactions between predictor variables. A summary description is given for the trend associated to a peak in occurrence probability for each regime. Smaller values indicate weaker interactions. All interactions were significant (*p* < 0.01). See the electronic supplementary material, figure S4 for the interaction plots. SST, sea surface temperature; max, maximum monthly climatological mean; STD, standard deviation of the long-term mean.

model	predictor 1	predictor 2	interaction size	summary
regime 1	complexity	non-commercial boat fishing	27.97	higher recreational boat fishing catch and lower complexity
	complexity	commercial fishing	27.76	higher commercial fishing catch and lower complexity
regime 2	wave_max	SST_STD	64.82	higher wave power and higher variation of sea surface temperature
	depth	wave_max	18.51	shallower depth and higher wave power
regime 3	irradiance_STD	SST_max	11.91	no clear pattern
	complexity	irradiance_max	11.47	no clear pattern
regime 5	irradiance_STD	invasive algae	25.35	lower variation of irradiance and observed presence of invasive algae
	depth	non-commercial boat fishing	15.55	deeper depth and higher recreational boat fishing

Regime 2 was best predicted by a strong positive relationship with maximum monthly climatological mean of wave power (36.5%), indicating a higher occurrence of this regime in wave-exposed sites (figure 3*b*). Cooler maximum monthly climatological sea surface temperature (9.3%), higher complexity (8.5%) and high variation of temperature (7.8%) all increased the probability for regime 2 to occur, while depth (8.7%) showed a slightly negative relationship. The two most important interactions (table 2) revealed a higher probability of occurrence as both wave power and temperature variation increased, and a weakening of the impact of waves at deeper depths (electronic supplementary material, figure S4b). The model explained 37% of the deviance and had an AUC score of 0.88.

Biophysical variables were also the most influential predictors of regime 3 (figure 3*c*). Occurrence probability was higher in places with low maximum monthly climatological chlorophyll-*a* concentration (13.6%)—but positively correlated with a higher frequency of anomalous chlorophyll-*a* events (7.7%). Regime 3 was more likely at depths shallower than 25 m (8.9%) and in wave-sheltered environments (8.2%). The interactions were weak, with no clear interaction pattern apparent (table 2; electronic supplementary material, figure S4c). The model explained 39% of the deviance and had an AUC score of 0.90.

Regime 5 was best predicted by depth (10.5%, peaked at mid-depth), increased topographical complexity of the seafloor (10.1%) and lower variation of irradiance (9.5%) (figure 3*d*). It was also associated with higher levels of new development along the coastline (9%) and, to a lesser extent, increased catch from non-commercial shore spearfishing (6.9%). The most important interaction involved variation of irradiance and observed presence of invasive algae (table 2). However, this result should be treated with caution owing to the scarcity of data and binary nature (i.e. presence only) of the invasive algae predictor. The second interaction was weaker and displayed a greater effect of recreational boat fishing with increasing depth (table 2; electronic supplementary material, figure S4d). The model explained 41% of the deviance and had an AUC score of 0.91.

### (c) Cross-scale patterns

Repeating the analysis at multiple standardized grain sizes (i.e. 500, 1000, 1500, 2500 and 4000 m) yielded largely similar results to the ones described above for all four regimes in terms of influential predictors and shape of the relationships. There was no significant difference across grain sizes with regard to model performance, or relative contribution of anthropogenic versus biophysical variables (electronic supplementary material, figure S5).

## 4. Discussion

Identifying the underlying drivers of different coral reef ecosystem regimes has great value for managers seeking viable strategies to avoid, or reverse, regime shifts. Drawing on an unprecedented compilation of data, this study presents, to our knowledge, the first attempt at quantifying the relative importance of anthropogenic and biophysical drivers in predicting reef ecosystems defined by both fish and benthic communities. As such, it offers novel insights into coral reef dynamics that can inform management strategies, as well as a promising analytical approach that might be applied in other ecosystems. Our findings provide empirical evidence that dealing with alternative regimes is inherently a social–ecological issue and that designing effective management interventions requires both focusing on prominent human drivers while accounting for the natural bounds set by the local biophysical environment.

The overwhelming influence of biophysical predictors in explaining the occurrence of three out of four regimes is striking. Only the most degraded regime, characterized by low fish biomass, few corals and high turf cover, was primarily predicted by anthropogenic variables (i.e. fishing and effluent). This confirms a large body of literature highlighting the detrimental effects of high fishing pressure and effluent discharge on reef ecosystems [31,32]. Studies have shown that fishing can disrupt coral reef trophic structures [33,34] and pave the way for algae to overgrow corals by removing key herbivores that would otherwise provide top-down algal control [10]. Similarly, excess nutrient delivery associated with local human populations has repeatedly been attributed to promoting the competitive abilities of algae [32,35], in particular turf algae [36].

Our findings also highlight the critical role of wave power and suggest that it drives the occurrence of a specific regime (i.e. regime 2), characterized by exposed sites with high fish biomass but limited coral cover. By contrast, regime 3, which displays substantial coral cover, occurs most commonly in sheltered environments with small pulses of chlorophyll-*a* in an otherwise rather oligotrophic background. This could illustrate how a pulsed delivery of oceanic-derived nutrients from physical processes such as internal waves or current-driven upwelling [37,38] may benefit corals on oligotrophic reefs by increasing ecosystem primary production and the energy available for coral growth [39].

Depth and complexity appeared almost systematically among the five most influential predictors, regardless of regime type. Both variables have been identified as key features influencing the structure of reef communities and offering potential for recovery from disturbances (electronic supplementary material, table S3). Areas with complex reef structure, for instance, provide refuge from predation and often harbour higher fish abundance and diversity [40]. While the most degraded regime (i.e. regime 1) was associated with very low complexity, the occurrence of regime 5, which supports diverse fish assemblages and high coral cover, peaked at mid-depth and increased with higher complexity. Depth and complexity also emerged as prominent interacting predictors, either weakening the effect of waves and favouring recreational boat fishing at deeper depths, or magnifying the impact of commercial fishing at low complexity. Our findings emphasize the value of these simple yet critical features in the management evaluation of a reef's resilience and clarify the mechanisms by which they can synergistically interact.

Defining ecological regimes allows capture of a considerable level of complexity of reef ecosystems [8,12]. The approach is also particularly appealing to managers who are often interested in the status of the reef as a whole, rather than its individual components. Yet, the descriptive advantage gained when merging multiple response variables may be counteracted by a reduction in the power to predict their occurrence, especially considering that species often exhibit individual and distinct responses to their surroundings. For instance, Gove *et al*. [16] improved model performance fivefold when moving from predicting the spatial variation in overall hard coral cover (11% deviance explained), to modelling the distribution of individual hard coral morphologies that show differential susceptibility to wave stress (55% deviance explained). While the regimes allow us to account for reciprocity between fish and benthic functional groups, they form a complex response variable made of organisms characterized by a wide range of attributes (e.g. slow versus fast growing, mobile versus sessile). Despite such heterogeneity, our models were able to consistently explain around 40% of the cross-validated deviance with high AUC values, thereby providing robust explanatory predictions of the mechanistic dynamics underlying ecological regimes.

Although different reef regimes were explained by a broad range of anthropogenic and biophysical variables, the particularly strong influence of the latter warrants further consideration. First, it may be specific to Hawaiian reefs. The archipelago is one of the most isolated in the world, is located at subtropical latitudes and experiences large oceanic forcings [18,19]. Some regimes might therefore be shaped by powerful biophysical drivers that supersede any human influence. Second, our findings could relate to the spatial scale of the analysis. Understanding the influence of scale requires analysing two major components: grain and extent. Grain refers to the finest spatial resolution within a given dataset, while extent relates to the overall area encompassed by the study [41]. While we were able to control for different grain sizes, we could not satisfyingly subset the data and run the BRTs for finer geographical areas than the Hawaiian Islands (e.g. a stretch of coastline) owing to sample size. This can obscure the relative importance of anthropogenic predictors that are likely to operate at the local level rather than at the regional-level, such as high sedimentation in an embayment [42]. Whether a stronger anthropogenic signature would emerge at finer scales of analysis, therefore, represents an important next step for future work that could better inform local community management. Finally, disentangling what represents anthropogenic and biophysical predictors can be difficult in an epoch where humans have become a dominant force in nature [43,44]. Rising seawater temperature, for instance, is profoundly influenced by human emissions of carbon dioxide into the atmosphere [45]. Similarly, nearshore chlorophyll-*a*, used here as a proxy for oceanic primary production [18], can also capture local aspects of water quality influenced by humans [38,46]. In addition, some biophysical conditions greatly influence anthropogenic impacts, such as large seasonal swell events preventing fishing activities, or flushing out sediment and effluent.

Coral reef managers are often faced with the challenge of where to allocate their limited resources and what management options to prioritize. Recent studies have shown the potential of fisheries regulations to facilitate reef recovery [47,48] and balance conservation objectives with stakeholders' interests [49]. Yet, less than one per cent of the coastline in the Hawaiian Islands is currently under no-take marine protected areas [50] and no licence is required for marine recreational fishing across the archipelago, although non-commercial catch has been estimated to be five times larger than commercial catch [51]. A growing tourism-based economy and planned development of new homes also have the potential to exacerbate pollution and runoff [52]. While our results provide additional evidence that addressing fishing pressure and water quality is critical to avoid degraded reef regimes, they also highlight which biophysical drivers need to be accounted for in a given location. There is little managers can do about broad-scale biophysical drivers, but understanding how environmental conditions shape coral reef regimes can help inform management strategies and identify priority areas. Importantly, our study provides the first step towards predicting the outcome of alternative management actions. By taking our results and turning them around for use in a forward-thinking model, future work should explore where change in a particular variable (or combination of variables) gives the quickest transition into a more desirable state. Such analysis would help identify where undesirable regimes may be naturally occurring and, otherwise, determine the most cost-effective management actions given a reef's natural setting.

In the wake of the 2014–2016 coral bleaching event, the State of Hawai‘i pledged to effectively manage 30 per cent of its nearshore waters by 2030. Our analyses, together with our publicly available database, represent valuable resources to assist managers and policy-makers in this process. Ultimately, however, addressing the challenges coral reefs are facing globally will also require identifying distal drivers of change (e.g. trade, climate change) and recognizing that leverage may lie far away from the reef [43,53]. Only through a combination of local and global management interventions, can we ensure coral reefs continue to provide the ecosystem services upon which so many people rely.

## Supplementary Material

Electronic Supplementary Material

## Data accessibility

Data are available from GitHub (https://github.com/JBjouffray/Hawaii_RegimesPredictors) and PacIOOS (http://www.pacioos.hawaii.edu/projects/oceantippingpoints/#data).
