# Supplementary material for: Parsing human and biophysical drivers of coral reef regimes
Source: Proc Biol Sci. 2019 Feb 13;286(1896):20182544. doi: 10.1098/rspb.2018.2544 (PMC6408596; doi:10.1098/rspb.2018.2544)
Supplement: Electronic Supplementary Material [file rspb20182544supp1.pdf]

ELECTRONIC SUPPLEMENTARY MATERIAL

Parsing human and biophysical drivers of coral reef regimes

Jean-Baptiste Jouffray[1,2*], Lisa M. Wedding[3], Albert V. Norström[1], Mary K. Donovan[4,5], Gareth J. Williams[6], Larry B. Crowder[3], Ashley L. Erickson[3], Alan M. Friedlander[4,7], Nicholas A.J. Graham[8], Jamison M. Gove[9], Carrie V. Kappel[10], John N. Kittinger[11,12], Joey Lecky[13,14], Kirsten L.L. Oleson[13], Kimberly A. Selkoe[10], Crow White[15], Ivor D. Williams[9], Magnus Nyström[1]

1 Stockholm Resilience Centre, Stockholm University, Stockholm, Sweden

2 Global Economic Dynamics and the Biosphere Academy Programme, Royal Swedish Academy of Sciences, Stockholm, Sweden

3 Hopkins Marine Station, Stanford University, Pacific Grove, CA 93950

4 Department of Biology, University of Hawai'i at Mānoa, Honolulu, HI, 96822

5 Hawai'i Institute of Marine Biology, University of Hawai'i at Mānoa, Kaneohe, HI 96744

6 School of Ocean Sciences, Bangor University, Anglesey, LL59 5AB, UK

7 Pristine Seas, National Geographic Society, Washington D.C, 20036

8 Lancaster Environment Centre, Lancaster University, Lancaster, LA1 4YQ, UK

9 Ecosystem Science Division, Pacific Islands Fisheries Science Center, National Oceanic Atmospheric Administration, Honolulu, HI, 96818

10 National Center for Ecological Analysis and Synthesis, University of California Santa Barbara, Santa Barbara, CA, 93101

11 Center for Oceans, Conservation International, Honolulu, HI, 96825

12 Julie Ann Wrigley Global Institute of Sustainability, Arizona State University, Tempe, AZ 85281

13 Department of Natural Resources and Environmental Management, University of Hawai'i at Mānoa, Honolulu, HI, 96822

14 Office of National Marine Sanctuaries, National Oceanic Atmospheric Administration, Honolulu, HI, 96818

15 Department of Biological Sciences, California Polytechnic State University, San Luis Obispo, CA, 93407

* corresponding author: jean-baptiste.jouffray@su.se

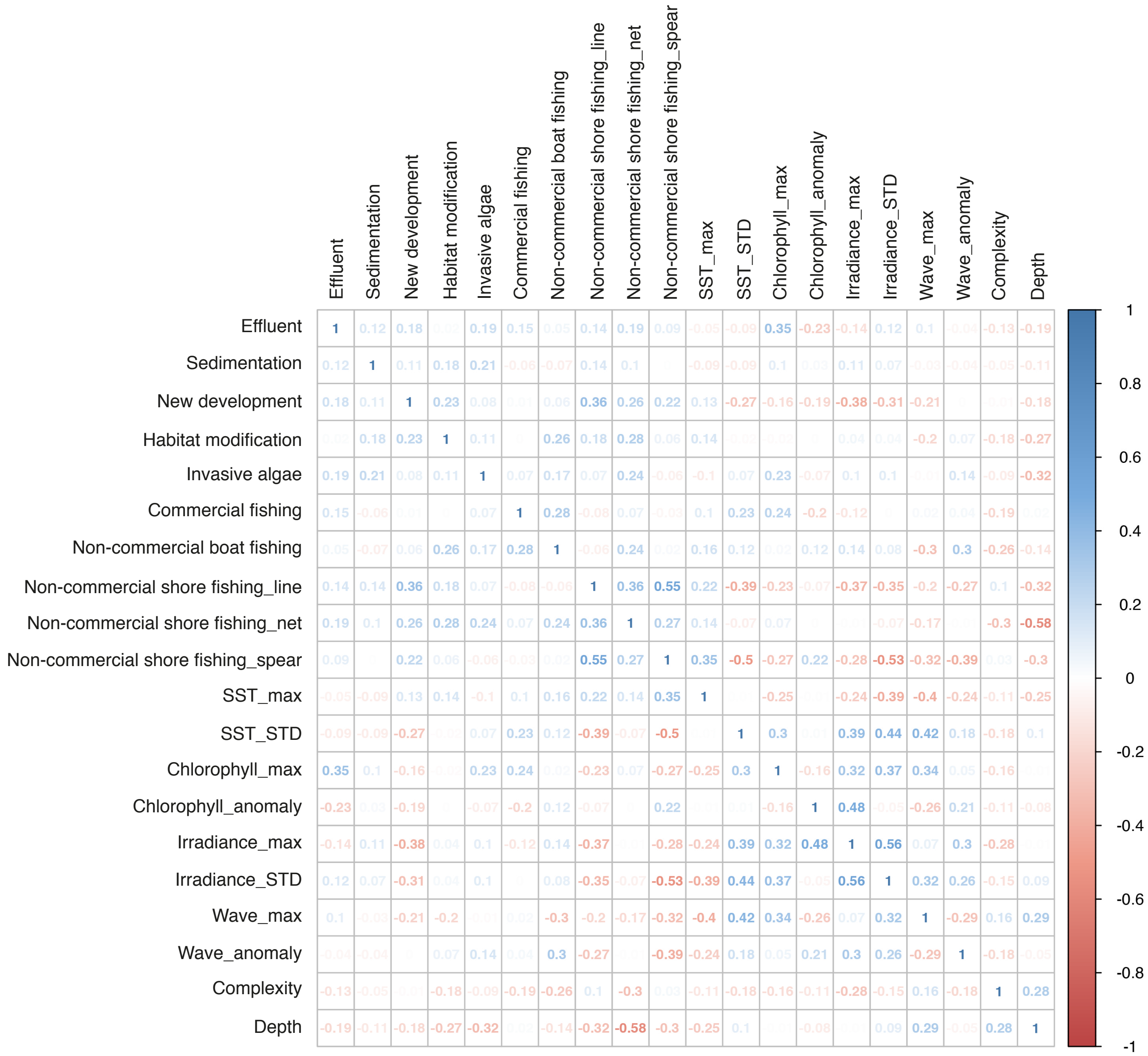

Figure S1. Collinearity matrix of all predictors used in the boosted regression trees analysis. See Table S2 for a description of each variable. SST: sea surface temperature; max: maximum monthly climatological mean; STD: standard deviation of the long-term mean; anomaly: frequency of anomalies.

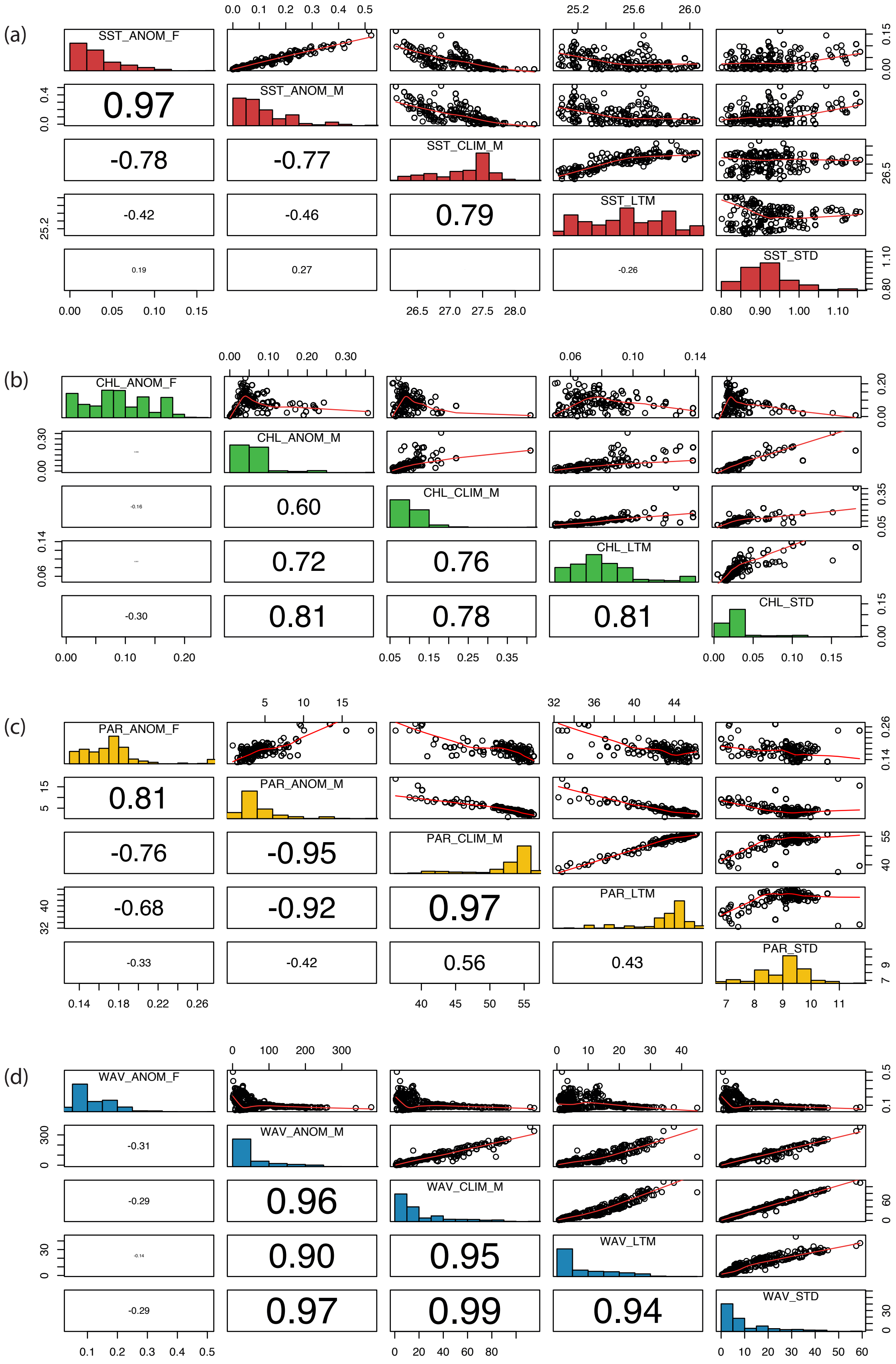

Figure S2. Correlation plots of environmental metrics for (a) sea surface temperature, (b) chlorophyll-*a*, (c) irradiance, and (d) wave power. ANOM_F: frequency of anomalies; ANOM_M: maximum of anomalies; CLIM_M: maximum monthly climatological mean; LTM: long-term mean; STD: standard deviation of the long-term mean.

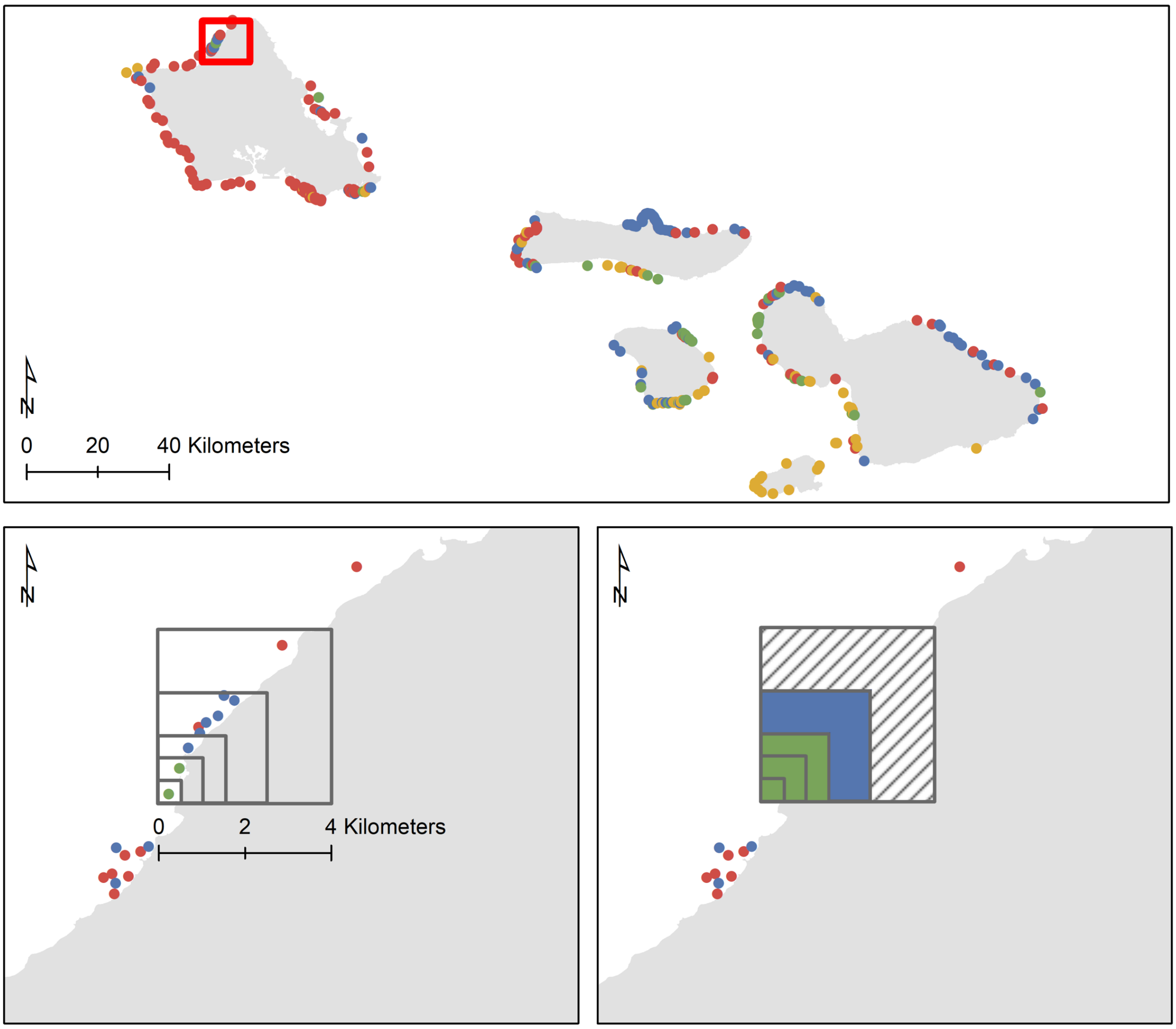

Figure S3. Schematic diagram illustrating the overall scaling approach used to address the potential influence of multiple spatial resolutions of predictor rasters. All predictors were extracted at multiple grain sizes (500m, 1000m, 1500m, 2500m, 4000m) and a two-thirds majority was applied to calculate the dominant regime for each grain size. This was performed by counting regimes within each cell and attributing to the cell the regime accounting for >66% of all sites within this cell. In case where a two-thirds majority could not be achieved, no regime was attributed to that cell (i.e., stripped area) and it was not included in the model. Otherwise, a new shapefile of points was created from coordinates based on the centre of the cell and used to extract all predictor variables from rasters at every grain sizes.

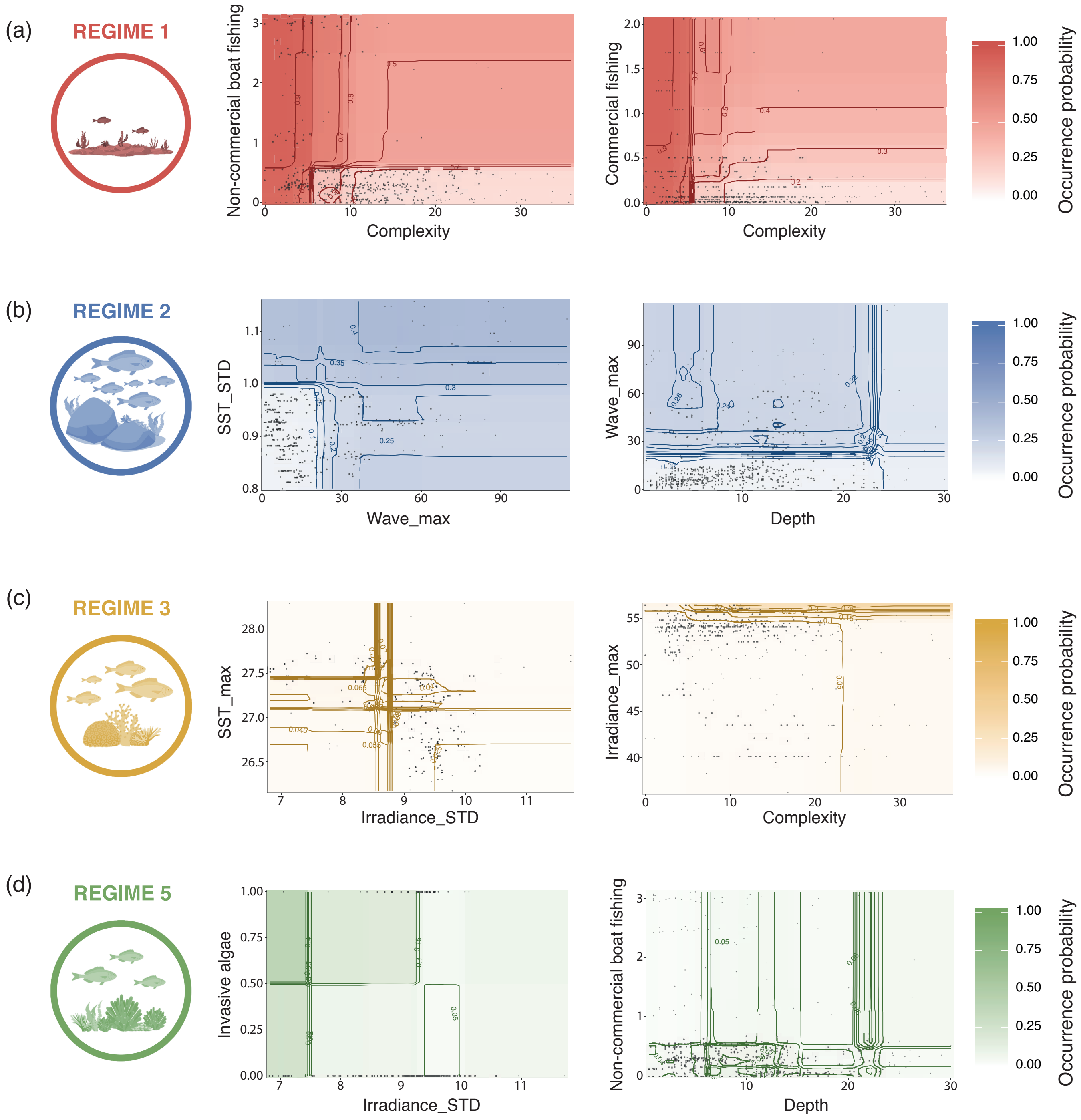

Figure S4. Interaction plots of the two strongest pairwise interactions between variables predicting the occurrence of each regime (a-d). Contour lines indicate values of occurrence probability and the dots represent observed data points. SST: sea surface temperature; max: maximum monthly climatological mean; STD: standard deviation of the long-term mean.

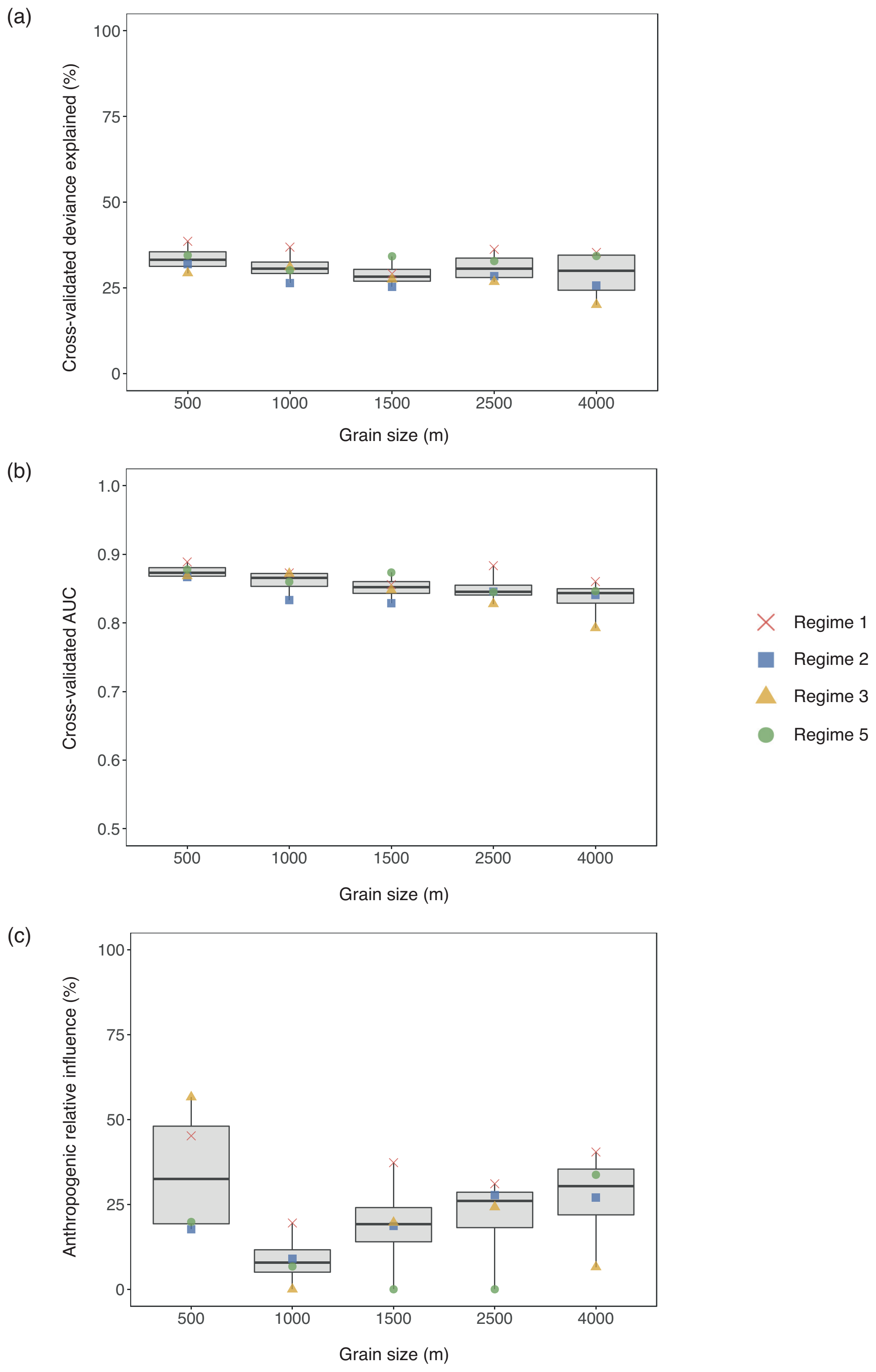

Figure S5. Cross-validated model performances (a-b) and relative anthropogenic influence (c) of boosted regression trees models run with predictor variables extracted at multiple standardized grain sizes (Figure S3). AUC: area under the receiver operating characteristic curve.

Table S1. Summary of the benthic and fish variables for each regime. Mean and 95% confidence intervals of the mean (± 2*standard error of mean) are reported. CCA stands for crustose coralline algae. Other includes sponges, sand, basalt rock and recently dead coral. Grazers, scrapers and browsers are all herbivorous fish. Grazers are known to prevent the establishment and growth of algae by cropping on algal turf. Scrapers also feed on turf but can remove components of the reef substratum as they feed which opens space for coral recruitment. Browsers consistently feed on macroalgae, playing a key role for reversing macroalgae-dominated reefs (Jouffray et al. 2015).

		**REGIME 1** (n=171)	**REGIME 2** (n=172)	**REGIME 3** (n=143)	**REGIME 5** (n=134)
BENTHIC COVER (%)	Coral	4.8 (± 1.1)	8.1 (± 1)	26.4 (± 3)	35.8 (± 2.2)
	CCA	3.0 (± 0.7)	7.0 (± 0.9)	6.9 (± 1.1)	8.1 (± 0.9)
	Turf algae	66.5 (± 3.1)	66.0 (± 2)	59.7 (± 3.4)	38.8 (± 2.2)
	Macroalgae	9.9 (± 1.8)	11.8 (± 1.5)	0 (± 0)	5.9 (± 1)
	Other	14.1 (± 2.3)	4.6 (± 0.9)	5.2 (± 1.4)	9.7 (± 1.6)
FISH BIOMASS (g m^{-2})	Grazers	4.6 (± 1.4)	30 (± 5.8)	17.6 (± 4.1)	10.1 (± 1.3)
	Scrapers	0.7 (± 0.3)	17.3 (± 3.9)	11.8 (± 2.8)	11.3 (± 1.8)
	Browsers	0.6 (± 0.3)	27.5 (± 9.7)	5.6 (± 2.1)	3.7 (± 0.8)
	Predators	0 (± 0)	11.9 (± 4.3)	9.2 (± 3.2)	4.5 (± 0.6)
	Secondary consumers	6.7 (± 1)	29.6 (± 4.5)	23.7 (± 2.9)	18.7 (± 1.6)

Table S2. Extended description of predictor variables used to explain the occurrence of multiple reef regimes. Raster data can be visualised in an online map viewer at: http://www.pacioos.hawaii.edu/projects/oceantippingpoints/#data.

	PREDICTOR	DESCRIPTION
Anthropogenic	Effluent	Nutrient run off (gallon/day/7 km^2) from onsite waste disposal systems (cesspools and septic tanks) measured by calculating the total flux within a 1.5 km radius of each map pixel based on sediment plume extents (7 km^2 ≈ area of a circle with 1.5 km radius)
	Sedimentation	Estimate of annual average amount of sediment (tons/year) delivered offshore as a function of vegetation cover characteristics, geologic substrate, soil erodibility, rainfall erosivity and slope, based on the Integrated Valuation of Ecosystem Services and Tradeoffs (InVEST) sediment delivery ratio model. Estimate sediment loads were aggregated by drainage basin to each point where a stream meets the coast and then dispersed offshore, resulting in a map of sediment plumes 1.5 km offshore
	New development	Relative level (0 to 1) of new development along the coastline based on image interpretation of the area that changed from undeveloped land to a hard, man-made surface between 2005 and 2011
	Habitat modification	Presence-absence of any alteration or removal of geomorphic structure as a result of human use. Includes artificial shoreline (e.g. seawalls, piers, breakwaters, filled wetlands), maintain channels and dredged areas, and offshore aquaculture infrastructure
	Invasive algae	Observed presence of any invasive algae (*Acanthophora spicifera, Gracilaria salicornia, Hypnea musciformis, Kappaphycus alvarezii*) synthesized from underwater visual surveys
	Commercial fishing	Annual average commercial reef fisheries catch (kg/ha) as reported in the State of Hawaiʻi Commercial Marine Landings Database by large irregular blocks ranging from 50-250km^2. Includes all gear types and no distinction between boat- and shore-based fishing
	Non-commercial boat fishing	Annual average non-commercial boat-based reef fisheries catch (kg/ha) from all gear types. Island means were distributed offshore by taking into consideration distance to harbour or launch ramps and human population within 30 km of the harbour or ramp
	Non-commercial shore fishing_line	Annual average non-commercial shore-based reef fisheries catch (kg/ha) by line. Island means were distributed offshore by taking into consideration shoreline accessibility (terrain steepness and presence of roads)
	Non-commercial shore fishing_net	Annual average non-commercial shore-based reef fisheries catch (kg/ha) by net. Island means were distributed offshore by taking into consideration shoreline accessibility (terrain steepness and presence of roads)
	Non-commercial shore fishing_spear	Annual average non-commercial shore-based reef fisheries catch (kg/ha) by spear. Island means were distributed offshore by taking into consideration shoreline accessibility (terrain steepness and presence of roads)
Biophysical	SST _max	Maximum monthly climatological mean of sea surface temperature (°C) quantified weekly from NOAA's satellite-derived observations
	SST_STD	Standard deviation of the long-term mean of weekly sea surface temperature (°C)
	Chlorophyll_max	Maximum monthly climatological mean of chlorophyll-*a* (mg/m^3) obtained from NASA's Moderate Resolution Imaging Spectroradiometer (MODIS) satellite observations (8-day composites)
	Chlorophyll_anomaly	Annual average of the total number of anomalous events for chlorophyll-*a,* represented as the percentage of time above the maximum monthly climatological mean
	Irradiance_max	Maximum monthly climatological mean of photosynthetically available radiation (Einstein/m^2/d) obtained from NASA's Moderate Resolution Imaging Spectroradiometer (MODIS) satellite observations (8-day composites)
	Irradiance_STD	Standard deviation of the long-term mean of 8-days irradiance composites (Einstein/m^2/d)
	Wave_max	Maximum monthly climatological mean of wave power (kW/m) obtained hourly using the University of Hawaiʻi Simulating Wave Nearshore (SWAN) model (Li et al. 2016)
	Wave_anomaly	Annual average of the total number of anomalous events for wave power, represented as the percentage of time above the maximum monthly climatological mean
	Complexity	Topographic complexity of the seafloor measured as slope of slope (i.e., the maximum rate of change in seafloor slope) using a gridded synthesis of multibeam sonar and Light Detection And Ranging (LiDAR) bathymetry (HMRG 2015)
	Depth	Depth of the seafloor in metres

Table S3. Relevance of predictors included in the analysis of the occurrence of multiple reef regimes defined by both fish and benthic functional groups.

PREDICTOR	RELEVANCE	REFERENCES
Effluent	Excess nutrients can influence water quality and promote rapid algal growth, outcompeting corals and disrupting the reef ecosystem. The State of Hawaiʻi has the highest number of onsite waste disposal systems (cesspools and septic tanks) per capita in the USA, many of which are adjacent to the coastline.	Kuntz et al 2005<br>Smith et al 2005<br>McClanahan et al 2007<br>Vermeij et al 2010<br>Wedding et al. 2018
Sedimentation	Sediment from various land-based stressors can affect reef health by smothering corals and blocking light, thereby impacting coral reproduction, growth rate, diversity, productivity, structure and life forms.	Rogers 1990<br>Fabricius 2005
New development	Areas of development and new construction sites can strip land of vegetation, leaving bare soil that is vulnerable to erosion. They often harbour additional large piles of soil on site for grading and landscaping which represent additional sources of sediment run-off.	Wedding et al 2018
Habitat modification	Habitat modification impacts coral reef environment through the alteration or removal of geomorphic structure as a result of human use.	Wedding et al. 2018
Invasive algae	Nonindigenous algae can pose a threat to biodiversity and alter the structure of reef ecosystems. Several alien species have become invasive in Hawaiʻi.	Smith et al. 2002<br>Wedding et al. 2018
Fishing catch	Fishing is a well-recognized driver of changing reef ecosystem conditions. Overfishing can reduce fish population which in turn influence benthic communities (e.g. algae growth control by herbivore fish). While distance and travel time to markets (i.e. commercial fishing) have been highlighted as strong predictors of fish assemblages on coral reefs globally, fishing in the main Hawaiian Islands is primarily recreational, with non-commercial fishing catch estimated to be five times larger than commercial catch.	Mumby et al. 2006<br>Williams et al. 2008<br>Cinner et al. 2013<br>Maire et al. 2016<br>McCoy et al. 2018
Sea surface temperature	Sea surface temperature plays an important role in a number of ecological processes occurring within coral reef environments. While low temperatures have been shown to limit coral growth, extended periods of high temperatures can lead to bleaching events with disastrous consequences for the reef.	Jokiel 1977<br>Hoegh-Guldberg 2007<br>Hughes et al. 2017
Chlorophyll-a	Chlorophyll-a is a widely-used proxy for phytoplankton biomass and an indicator for changes in primary production. Phytoplankton biomass drives marine ecosystem trophic structure, influencing both fisheries productivity and benthic dynamics. It is particularly influential near coral reef islands and atolls, where nearshore phytoplankton can favour calcifying organisms, although excessive concentration may as well enhance fleshy algal growth.	Duarte and Cebrian 1996<br>Chassot et al 2010<br>Gove et al. 2016
Irradiance	Irradiance represents the amount of solar radiation (sunlight) at the ocean surface that is available for photosynthesis. Solar radiation is the main external source of energy for marine ecosystems and can limit the depth and range of coral growth.	Kleypas et al. 1999<br>Chassot et al. 2010<br>Gove et al. 2013
Wave power	Wave power incorporates both wave period and wave height, thereby representing a more realistic estimate of wave-induced stress on coral reefs, which has been shown to filter fish assemblages and influence benthic distribution patterns and morphology.	Dollar 1982<br>Friedlander et al. 2003<br>Williams et al. 2013<br>Gove et al. 2013, 2015<br>Bejarano et al. 2017
Complexity	Areas of complex reef structure provide refuge from predation and often harbour higher fish biomass and density. Structural complexity has been identified as a key factor determining spatial patterns of herbivory and functional structure of reef fish assemblages.	Wedding et al. 2008<br>Vergés et al. 2011<br>Graham and Nash 2013<br>Rogers et al. 2014<br>Darling et al. 2017<br>Richardson et al. 2017
Depth	Due to differences in light penetration and vulnerability to change, depth strongly influences the distribution of both benthic organisms and fish assemblages. It provides refuge in the face of bleaching events, stratifies coral trophic zonation and constitutes a key factor of potential reef recovery.	Friedlander and Parrish 1998<br>Williams et al. 2013<br>Graham et al. 2015<br>Muir et al. 2017<br>Williams et al. 2018

Table S4. Variance inflation factor (VIF) scores for all predictors included in the boosted regression trees analysis. See Table 1 for a description of each variable. SST: sea surface temperature; max: maximum monthly climatological mean; STD: standard deviation of the long-term mean; anomaly: frequency of anomalies.

PREDICTOR	VIF
Effluent	1.65
Sedimentation	1.25
New development	1.48
Habitat modification	1.32
Invasive algae	1.32
Commercial fishing	1.49
Non-commercial boat fishing	1.65
Non-commercial shore fishing_line	1.86
Non-commercial shore fishing_net	1.82
Non-commercial shore fishing_spear	2.97
SST_max	2.26
SST_STD	3.09
Chlorophyll_max	1.98
Chlorophyll_anomaly	2.40
Irradiance_max	3.28
Irradiance_STD	2.43
Wave_max	2.94
Wave_anomaly	2.21
Complexity	1.42
Depth	2.10

Table S5. Summary of model performances and spatial autocorrelation. As expected, the model performances assessed on training data (used for model fitting) were higher than the cross-validated (cv) ones (based on left out data). The former indicates how good the model is at explaining observed data while the later tells how good the model is at predicting new data (Buston and Elith 2011). Moran'I is a measure of spatial autocorrelation that ranges from -1 to 1, with values close to zero indicating no spatial autocorrelation. AUC: area under the receiver operating characteristic curve.

	REGIME 1	REGIME 2	REGIME 3	REGIME 5
Number of trees	4950	3350	5850	8200
Total deviance	1.18	1.18	1.08	1.04
Residual deviance	0.42	0.52	0.37	0.28
AUC	0.98	0.96	0.98	1.00
Deviance explained (%)	64.2	55.6	65.5	73.5
cv deviance	0.69	0.75	0.65	0.62
cv AUC	0.90	0.88	0.90	0.91
cv deviance explained (%)	41.0	36.5	39.4	41.0
Moran's I	0.04	0.04	0.03	0.02

REFERENCES

Bejarano, S., Jouffray, J.B., Chollett, I., Allen, R., Roff, G., Marshell, A., Steneck, R., Ferse, S.C. and Mumby, P.J., 2017. The shape of success in a turbulent world: wave exposure filtering of coral reef herbivory. *Functional Ecology*, *31*(6), pp.1312-1324.

Buston, P.M. and Elith, J., 2011. Determinants of reproductive success in dominant pairs of clownfish: a boosted regression tree analysis. *Journal of Animal Ecology*, *80*(3), pp.528-538.

Chassot, E., Bonhommeau, S., Dulvy, N.K., Mélin, F., Watson, R., Gascuel, D. and Le Pape, O., 2010. Global marine primary production constrains fisheries catches. *Ecology letters*, *13*(4), pp.495-505.

Cinner, J.E., Graham, N.A., Huchery, C. and MacNeil, M.A., 2013. Global effects of local human population density and distance to markets on the condition of coral reef fisheries. *Conservation Biology*, *27*(3), pp.453-458.

Darling, E.S., Graham, N.A., Januchowski-Hartley, F.A., Nash, K.L., Pratchett, M.S. and Wilson, S.K., 2017. Relationships between structural complexity, coral traits, and reef fish assemblages. *Coral Reefs*, *36*(2), pp.561-575.

Dollar, S.J., 1982. Wave stress and coral community structure in Hawaii. *Coral Reefs*, *1*(2), pp.71-81.

Duarte, C.M. and Cebrian, J., 1996. The fate of marine autotrophic production. *Limnology and Oceanography*, *41*(8), pp.1758-1766.

Fabricius, K.E., 2005. Effects of terrestrial runoff on the ecology of corals and coral reefs: review and synthesis. *Marine Pollution Bulletin*, *50*, pp.125-146.

Friedlander, A.M. and Parrish, J.D., 1998. Habitat characteristics affecting fish assemblages on a Hawaiian coral reef. *Journal of Experimental Marine Biology and Ecology*, *224*(1), pp.1-30.

Friedlander, A.M., Brown, E.K., Jokiel, P.L., Smith, W.R. and Rodgers, K.S., 2003. Effects of habitat, wave exposure, and marine protected area status on coral reef fish assemblages in the Hawaiian archipelago. *Coral Reefs*, *22*(3), pp.291-305.

Gove, J.M., Williams, G.J., McManus, M.A., Heron, S.F., Sandin, S.A., Vetter, O.J. and Foley, D.G., 2013. Quantifying climatological ranges and anomalies for Pacific coral reef ecosystems. *PloS one*, *8*(4), p.e61974.

Gove, J.M., Williams, G.J., McManus, M.A., Clark, S.J., Ehses, J.S. and Wedding, L.M., 2015. Coral reef benthic regimes exhibit non-linear threshold responses to natural physical drivers. *Marine Ecology Progress Series*, *522*, pp.33-48.

Gove, J.M., McManus, M.A., Neuheimer, A.B., Polovina, J.J., Drazen, J.C., Smith, C.R., Merrifield, M.A., Friedlander, A.M., Ehses, J.S., Young, C.W. and Dillon, A.K., 2016. Near-island biological hotspots in barren ocean basins. *Nature communications*, *7*, p.10581.

Graham, N.A.J. and Nash, K.L., 2013. The importance of structural complexity in coral reef ecosystems. *Coral Reefs*, *32*(2), pp.315-326.

Graham, N.A., Jennings, S., MacNeil, M.A., Mouillot, D. and Wilson, S.K., 2015. Predicting climate-driven regime shifts versus rebound potential in coral reefs. *Nature*, *518*(7537), pp.94-97.

HMRG. 2015 Hawai‘i Mapping Research Group. Hawai‘i Mapping Research Group, School of Ocean and Earth Science and Technology, University of Hawai‘i at Manoa. See http://www.soest.hawaii.edu/HMRG/cms/ (accessed on 8 June 2016).

Hoegh-Guldberg, O., Mumby, P.J., Hooten, A.J., Steneck, R.S., Greenfield, P., Gomez, E., Harvell, C.D., Sale, P.F., Edwards, A.J., Caldeira, K. and Knowlton, N., 2007. Coral reefs under rapid climate change and ocean acidification. *science*, *318*(5857), pp.1737-1742.

Hughes, T.P., Kerry, J.T., Álvarez-Noriega, M., Álvarez-Romero, J.G., Anderson, K.D., Baird, A.H., Babcock, R.C., Beger, M., Bellwood, D.R., Berkelmans, R. and Bridge, T.C., 2017. Global warming and recurrent mass bleaching of corals. *Nature*, *543*(7645), p.373.

Jokiel, P.L. and Coles, S.L., 1977. Effects of temperature on the mortality and growth of Hawaiian reef corals. *Marine Biology*, *43*(3), pp.201-208.

Jouffray, J.B., Nyström, M., Norström, A.V., Williams, I.D., Wedding, L.M., Kittinger, J.N. and Williams, G.J., 2015. Identifying multiple coral reef regimes and their drivers across the Hawaiian archipelago. *Philosophical Transactions of the Royal Society of London B: Biological Sciences*, *370*(1659), p.20130268.

Kleypas, J.A., McManus, J.W. and Menez, L.A., 1999. Environmental limits to coral reef development: where do we draw the line?. *American Zoologist*, *39*(1), pp.146-159.

Kuntz, N.M., Kline, D.I., Sandin, S.A. and Rohwer, F., 2005. Pathologies and mortality rates caused by organic carbon and nutrient stressors in three Caribbean coral species. *Marine Ecology Progress Series*, *294*, pp.173-180.

Li N, Cheung KF, Stopa JE, Hsiao F, Chen YL, Vega L, Cross P. 2016 Thirty-four years of Hawaii wave hindcast from downscaling of climate forecast system reanalysis. Ocean Model. 100, 78–95. (doi:10.1016/j.ocemod.2016.02.001)

Maire, E., Cinner, J., Velez, L., Huchery, C., Mora, C., Dagata, S., Vigliola, L., Wantiez, L., Kulbicki, M. and Mouillot, D., 2016. How accessible are coral reefs to people? A global assessment based on travel time. *Ecology letters*, *19*(4), pp.351-360.

McClanahan, T.R., Carreiro-Silva, M. and DiLorenzo, M., 2007. Effect of nitrogen, phosphorous, and their interaction on coral reef algal succession in Glover's Reef, Belize. *Marine Pollution Bulletin*, *54*(12), pp.1947-1957.

McCoy KS, Williams ID, Friedlander AM, Ma H, Teneva L, Kittinger JN. 2018. Estimating nearshore coral reef-associated fisheries production from the main Hawaiian Islands. *PLoS ONE* 13(4): e0195840.

Muir, P.R., Marshall, P.A., Abdulla, A. and Aguirre, J.D., 2017. Species identity and depth predict bleaching severity in reef-building corals: shall the deep inherit the reef?. *Proc. R. Soc. B*, *284*(1864), p.20171551.

Mumby, P.J., Dahlgren, C.P., Harborne, A.R., Kappel, C.V., Micheli, F., Brumbaugh, D.R., Holmes, K.E., Mendes, J.M., Broad, K., Sanchirico, J.N. and Buch, K., 2006. Fishing, trophic cascades, and the process of grazing on coral reefs. *Science*, *311*(5757), pp.98-101.

Richardson, L.E., Graham, N.A., Pratchett, M.S. and Hoey, A.S., 2017. Structural complexity mediates functional structure of reef fish assemblages among coral habitats. *Environmental Biology of Fishes*, *100*(3), pp.193-207.

Rogers, C.S., 1990. Responses of coral reefs and reef organisms to sedimentation. *Marine ecology progress series*, pp.185-202.

Rogers, A., Blanchard, J.L. and Mumby, P.J., 2014. Vulnerability of coral reef fisheries to a loss of structural complexity. *Current Biology*, *24*(9), pp.1000-1005.

Smith, J.E., Hunter, C.L. and Smith, C.M., 2002. Distribution and reproductive characteristics of nonindigenous and invasive marine algae in the Hawaiian Islands. *Pacific Science*, *56*(3), pp.299-315.

Smith, J.E., Runcie, J.W. and Smith, C.M., 2005. Characterization of a large-scale ephemeral bloom of the green alga Cladophora sericea on the coral reefs of West Maui, Hawai'i. *Marine Ecology Progress Series*, *302*, pp.77-91.

Vergés, A., Vanderklift, M.A., Doropoulos, C. and Hyndes, G.A., 2011. Spatial patterns in herbivory on a coral reef are influenced by structural complexity but not by algal traits. *PloS one*, *6*(2), p.e17115.

Vermeij, M.J., Van Moorselaar, I., Engelhard, S., Hörnlein, C., Vonk, S.M. and Visser, P.M., 2010. The effects of nutrient enrichment and herbivore abundance on the ability of turf algae to overgrow coral in the Caribbean. *PloS one*, *5*(12), p.e14312.

Wedding, L.M., Friedlander, A.M., McGranaghan, M., Yost, R.S. and Monaco, M.E., 2008. Using bathymetric lidar to define nearshore benthic habitat complexity: Implications for management of reef fish assemblages in Hawaii. *Remote Sensing of Environment*, *112*(11), pp.4159-4165.

Wedding, L.M., Lecky, J., Gove, J.M., Walecka, H.R., Donovan, M.K., Williams, G.J., Jouffray, J.B., Crowder, L.B., Erickson, A., Falinski, K. and Friedlander, A.M., 2018. Advancing the integration of spatial data to map human and natural drivers on coral reefs. *PloS one*, *13*(3), p.e0189792.

Williams, I.D., Walsh, W.J., Schroeder, R.E., Friedlander, A.M., Richards, B.L. and Stamoulis, K.A., 2008. Assessing the importance of fishing impacts on Hawaiian coral reef fish assemblages along regional-scale human population gradients. *Environmental Conservation*, *35*(3), pp.261-272.

Williams, G.J., Smith, J.E., Conklin, E.J., Gove, J.M., Sala, E. and Sandin, S.A., 2013. Benthic communities at two remote Pacific coral reefs: effects of reef habitat, depth, and wave energy gradients on spatial patterns. *PeerJ*, *1*, p.e81.

Williams, G.J., Sandin, S.A., Zgliczynski, B.J., Fox, M.D., Gove, J.M., Rogers, J.S., Furby, K.A., Hartmann, A.C., Caldwell, Z.R., Price, N.N. and Smith, J.E., 2018. Biophysical drivers of coral trophic depth zonation. *Marine Biology*, *165*(4), p.60.
